# Case Report: Prenatal ultrasound presentation of congenital melanocytic nevus syndrome

**DOI:** 10.3389/fped.2024.1466999

**Published:** 2024-12-24

**Authors:** ZhiH Shi, TingT Sun, Juan Yin, Shuo Qiu, YueM Wang, JunH Leng

**Affiliations:** Department of Ultrasound, Jinan Maternity and Child Care Hospital, Jinan, Shandong, China

**Keywords:** prenatal ultrasound, congenital melanocytic nevus syndrome, cerebellar, amygdaloid complex, posterior fossa cyst

## Abstract

Congenital melanocytic nevus (CMN) syndrome is a rare, non-familial neural ectodermal dysplasia characterized by CMN combined with extracutaneous abnormalities, predominantly involving the central nervous system (CNS). The pathogenesis of CMN syndrome is thought to result from early post-zygotic somatic mutations. CNS melanosis frequently affects the anterior temporal lobes, brainstem, cerebellum, and cerebral cortex. Magnetic resonance imaging typically demonstrates T1 hyperintensity associated with CNS melanosis, while ultrasound often reveals abnormal echogenicity. We report a case of a fetus diagnosed with CMN syndrome, presenting with abnormal echogenicity in the cerebellar and amygdaloid complexes and a posterior fossa cyst. Autopsy identified two melanocytic nevi on the lumbosacral region of the fetus. Reports linking CMN syndrome to fetal intracranial abnormalities remain exceedingly rare.

## Introduction

Congenital melanocytic nevus (CMN) syndrome is a rare, non-familial neural ectodermal dysplasia characterized by CMN accompanied by extracutaneous abnormalities, primarily affecting the central nervous system (CNS) (1, 2). Its pathogenesis is attributed to early post-zygotic somatic mutations arising from mosaicism involving heterozygous activating mutations at codon 61 of the *NRAS* gene (neuroblastoma RAS viral oncogene homolog), a critical developmental gene regulating key cell signaling pathways (3).

Although histological findings remain the diagnostic gold standard, neuroimaging and clinical features are instrumental in suspecting CMN syndrome (4). In 2012, Kinsler et al. proposed diagnostic criteria for CMN syndrome, including (a) the presence of a CMN with an anticipated adult size exceeding 5 cm or multiple CMNs of any size at birth and (b) neurological involvement (clinical or radiological) and/or three or more characteristic facial features (5).

CNS abnormalities in CMN syndrome frequently involve the leptomeninges, occasionally extending into brain parenchyma. The cerebellum and anterior temporal lobes are the most common sites of melanocytic deposition (6). CNS melanosis typically appears hyperintense on T1-weighted magnetic resonance imaging (MRI) due to the paramagnetic properties of melanin (7) and shows abnormal echogenicity on ultrasound (8). Common neurological manifestations include increased intracranial pressure, seizures, and neurodevelopmental delay (2, 7). The prognosis for individuals with neurological symptoms of CMN syndrome is extremely poor.

## Case report

**Figure 1:**
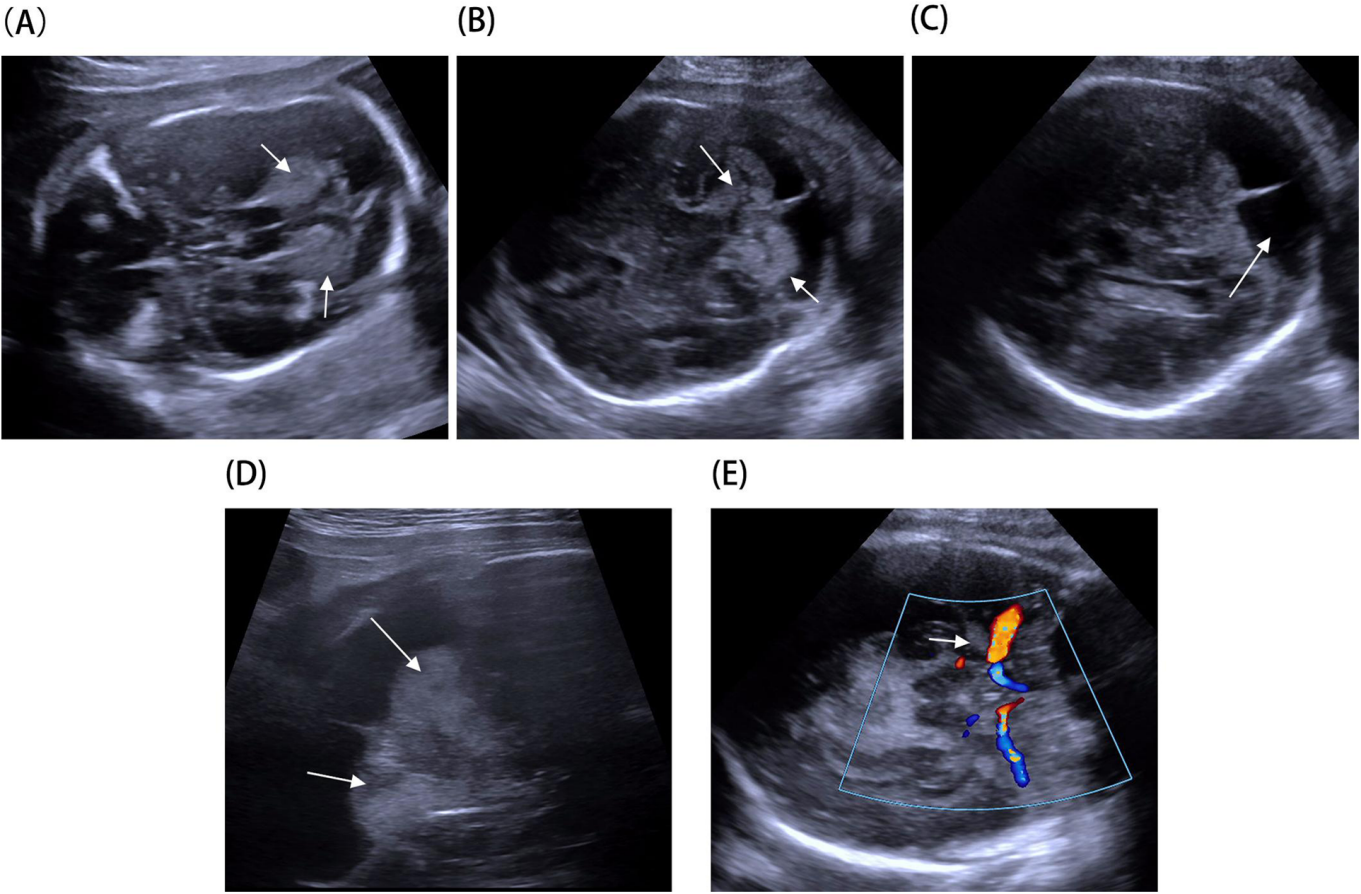
Fetal brain ultrasound imaging: **(A)** hyperechoic cerebellar alterations observed at 24 weeks (arrow). **(B)** Hyperechoic cerebellar alterations observed at 28 weeks (arrow). **(C)** Posterior fossa cyst identified at 28 weeks (arrow). **(D)** Hyperechoic cerebellar alterations at 32 weeks using a high-frequency probe (arrow). **(E)** Hyperechoic alterations in the amygdaloid complex at 32 weeks (arrow).

**Figure 2:**
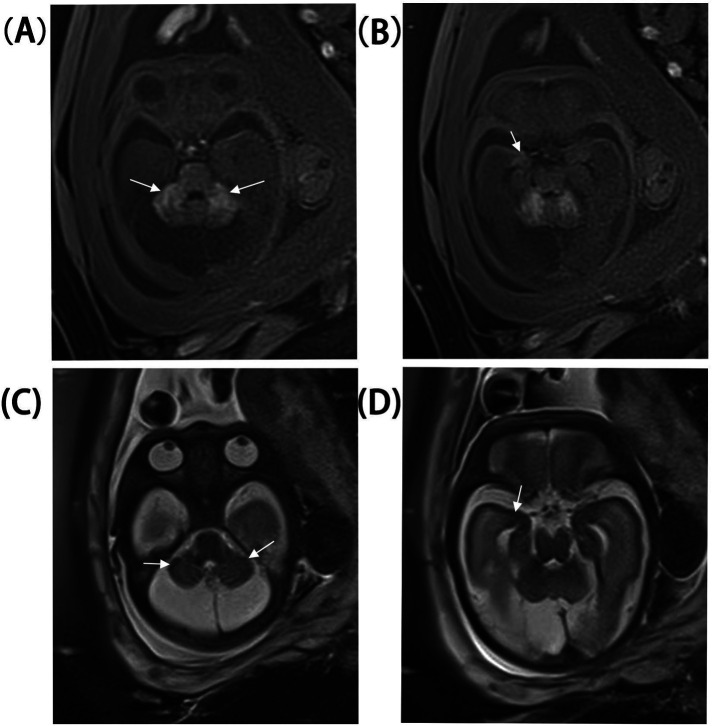
Fetal brain MRI at 30 weeks. **(A,B)** Cerebellum and amygdaloid complex T1 shortening. **(C,D)** Cerebellum and amygdaloid complex T2 shortening.

A 33-year-old pregnant woman underwent a fetal ultrasound at 24 weeks of gestation, revealing diffuse hyperechogenicity in the cerebellum with indistinct fissures on the cerebellar surface (Figure 1A). The posterior fossa measured approximately 5 mm in width. At 28 weeks, a follow-up ultrasound of the cerebellum (Figure 1B) showed no significant changes compared to the 24-week scan. However, the posterior fossa had expanded to approximately 11 mm, and a posterior fossa cyst was identified (Figure 1C). By 32 weeks, persistent cerebellar abnormalities and the posterior fossa cyst remained visible (Figure 1D), and abnormal echogenicity was observed within the amygdaloid complex (Figure 1E). Fetal brain MRI revealed T1 shortening in both the cerebellum and amygdaloid complex (Figure 2A,B), along with T2 shortening (Figures 2C,D). In light of these findings, the parents opted for pregnancy termination. Autopsy findings identified two melanocytic nevi located on the fetus's lumbosacral region.

## Discussion

The term “neurocutaneous melanocytosis” or “neurocutaneous melanosis” (NCM) has historically been used to describe neurological abnormalities associated with multiple CMN, ranging from benign melanocyte proliferations to melanoma. Given the evolving and inconsistent definitions of NCM across publications, the term “CMN syndrome” has been proposed to encompass CMN with any extracutaneous system involvement (1). Accordingly, this report adopts the term CMN syndrome.

Melanocytes, specialized cells responsible for melanin production and storage, originate from the neural crest and migrate to various tissues, including the skin, mucosa, uveal ocular layer, inner ear, and CNS between the 8th and 10th weeks of embryonic development. Within the CNS, melanocytes are primarily located in the leptomeninges, with preferential distribution at the skull base convexity, ventral brainstem, and cervical cord (9, 10). This migration occurs along autonomic and sensory nerves, vascular structures, and adnexal pathways, explaining both the infiltration of melanin-containing cells into the nervous system and the perivascular involvement observed in benign congenital nevi and CNS lesions in CMN syndrome (6).

CMN syndrome can affect the CNS, presenting with or without symptoms. CNS involvement primarily manifests as progressive hydrocephalus due to leptomeningeal melanocytic infiltration and may include signs of intracranial hypertension, seizures, or cranial nerve dysfunction (2, 7). Affected children generally have a poor prognosis, with most succumbing within 3 years of symptom onset.

In this case, fetal brain MRI demonstrated T1 shortening in the cerebellum and amygdaloid complex, as well as T2 shortening, consistent with typical imaging findings of CNS melanosis. MRI is the preferred imaging modality for CMN syndrome. Melanosis is most easily detected on T1-weighted images before myelination. Melanotic brain lesions typically appear as T1 hyperintense and T2 hypointense relative to normal immature brain tissue due to the paramagnetic properties of stable free radicals in melanin pigment (11). The most common sites of cerebral melanosis are the anterior amygdala, brainstem, cerebellum, and cerebral cortex (7). In addition, hindbrain malformations, such as small or dysmorphic cerebellar hemispheres or inferior vermian hypoplasia, are significantly associated with concurrent hindbrain melanosis.

Ultrasound is another widely used imaging method for evaluating infant brains. While reports of brain melanosis identified via ultrasound are rare, Chen et al. documented a case involving a male infant with small echogenic foci in the left thalamus and left choroidal fissure (8). Only one prior prenatal ultrasound report concerning NCM has been documented, showing a cystic lesion in the posterior fossa (12). Posterior fossa cysts are thought to arise from impaired cerebrospinal fluid (CSF) absorption due to melanocytic deposits along the leptomeninges of the cerebellum.

In this case, hyperechoic cerebellar lesions were observed during the mid-trimester, while bilateral hyperechoic lesions in the amygdaloid complex were noted in the third trimester. Hyperechoic cerebellar findings often suggest hemorrhage, and their echogenicity may evolve with disease progression. MRI findings revealed T1 hyperintensity and T2 hypointensity, characteristic of CMN syndrome. Autopsy identified two melanocytic nevi on the lumbosacral region of the fetus. Based on the diagnostic criteria for CMN syndrome proposed by Kinsler et al. (5), a diagnosis of CMN syndrome was established for this fetus.

This case expands the spectrum of prenatal presentations of CMN syndrome by describing high-echo abnormalities in the cerebellar and amygdaloid complexes. Given the absence of specific imaging criteria for prenatal diagnosis, CMN syndrome should be considered in the differential diagnosis of high-echo findings in the cerebellar and amygdaloid complexes on fetal imaging. In addition, T1-weighted imaging during fetal MRI should be prioritized to enhance the detection of CNS melanin deposits.

## Data Availability

The original contributions presented in the study are included in the article/Supplementary Material, further inquiries can be directed to the corresponding author.

## References

[1] Waelchli R Aylett SE Atherton D Thompson DJ Chong WK Kinsler VA. Classification of neurological abnormalities in children with congenital melanocytic naevus syndrome identifies magnetic resonance imaging as the best predictor of clinical outcome. Br J Dermatol. (2015) 173(3):739–50. 10.1111/bjd.13898 25966033 PMC4737261

[2] Ruth J. Congenital melanocytic nevus syndrome: an association between congenital melanocytic nevi and neurological abnormalities. Semin Pediatr Neurol. (2024) 51:101153. 10.1016/j.spen.2024.101153 39389659

[3] Guler E Arslan EA. A case of neurocutaneous melanosis and neuroimaging findings. Radiol Case Rep. (2015) 9(3):1–6. 10.3941/jrcr.v9i3.2141 25926927 PMC4395010

[4] Mormina E Granata F Vinci SL Coglitore A Caragliano AA Tessitore A Imaging and clinical features of neurocutaneous melanosis in the pediatric population. Curr Med Imaging. (2021) 17(12):1391–402. 10.2174/1573405617666210527091109 34047260

[5] Kinsler V Shaw AC Merks JH Hennekam RC. The face in congenital melanocytic nevus syndrome. Am J Med Genet A. (2012) 158A(5):1014–9. 10.1002/ajmg.a.34217 22438093

[6] Barkovich AJ Frieden IJ Williams ML. MR of neurocutaneous melanosis. AJNR Am J Neuroradiol. (1994) 15(5):859–67. 8059652 PMC8332186

[7] Jakchairoongruang K Khakoo Y Beckwith M Barkovich AJ. New insights into neurocutaneous melanosis. Pediatr Radiol. (2018) 48(12):1786–96. 10.1007/s00247-018-4205-x 30074086 PMC7469866

[8] Chen YA Woodley-Cook J Sgro M Bharatha A. Sonographic and magnetic resonance imaging findings of neurocutaneous melanosis. Radiol Case Rep. (2016) 11(1):29–32. 10.1016/j.radcr.2015.12.004 26973729 PMC4769615

[9] Küsters-Vandevelde HV Küsters B van Engen-van Grunsven AC Groenen PJ Wesseling P Blokx WA. Primary melanocytic tumors of the central nervous system: a review with focus on molecular aspects. Brain Pathol. (2015) 25(2):209–26. 10.1111/bpa.12241 25534128 PMC8029366

[10] Varela-Poblete J Vidal-Tellez A Cruz-Quiroga JP Montoya-Salvadores F Medina-Escobar J. Melanocytic lesions of the central nervous system: a case series. Arq Neuropsiquiatr. (2022) 80(2):153–60. 10.1590/0004-282x-anp-2021-0082 35352754 PMC9648915

[11] Enochs WS Petherick P Bogdanova A Mohr U Weissleder R. Paramagnetic metal scavenging by melanin: MR imaging. Radiology. (1997) 204(2):417–23. 10.1148/radiology.204.2.9240529 9240529

[12] East JE Soares BP. Neurocutaneous melanosis: prenatal presentation as a posterior Fossa cyst. Neuropediatrics. (2021) 52(6):504–5. 10.1055/s-0040-1722676 33445188

