# Supplementary figures and images for: Case Report: Prenatal ultrasound presentation of congenital melanocytic nevus syndrome
Source: Front Pediatr. 2024 Dec 24;12:1466999. doi: 10.3389/fped.2024.1466999 (PMC11703949; doi:10.3389/fped.2024.1466999)

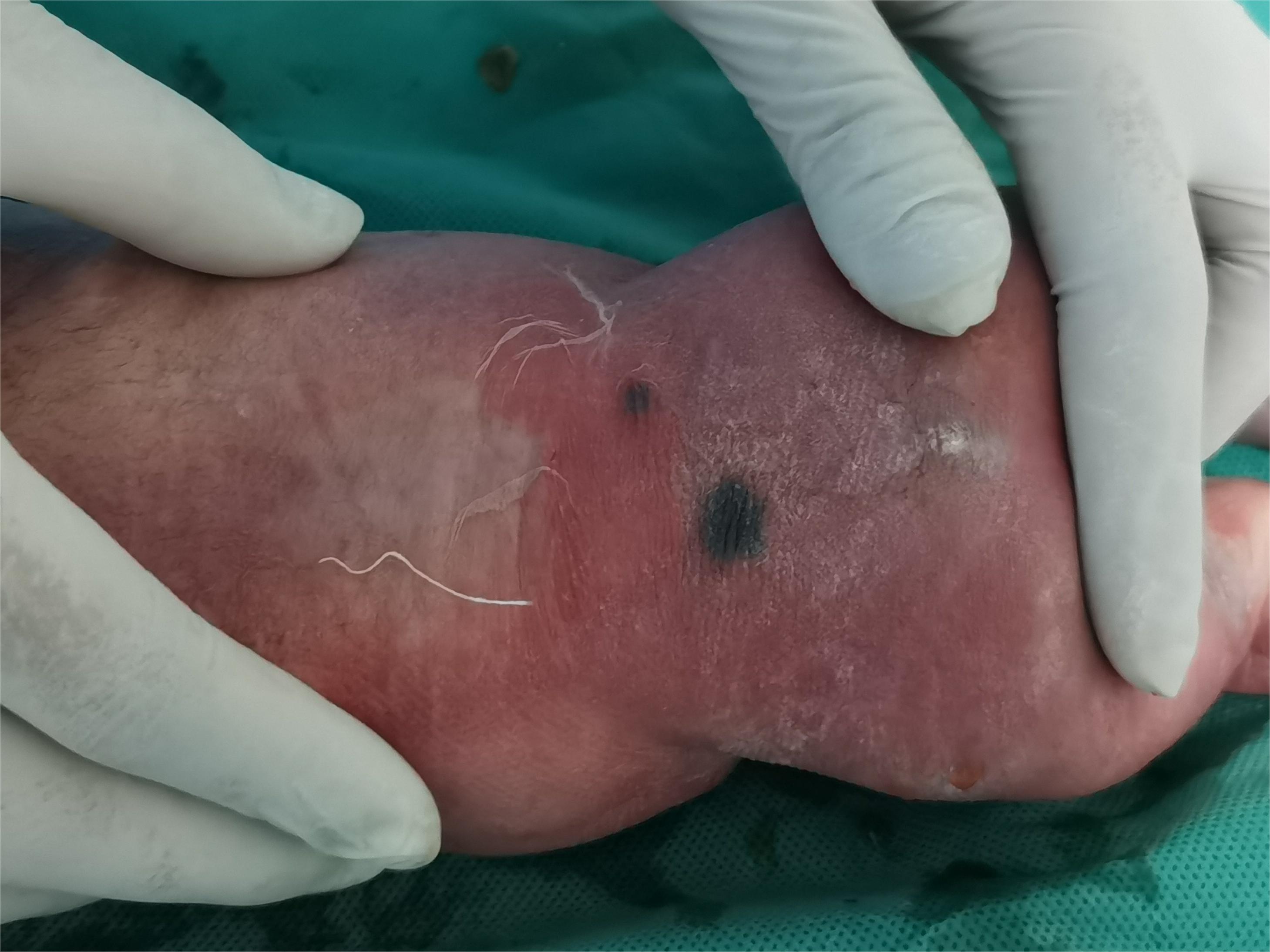

Supplement: Supplementary file 1 [file Image1.jpeg]
